# Optimism and Mental Health of Minority Students: Moderating Effects of Cultural Adaptability

**DOI:** 10.3389/fpsyg.2019.02545

**Published:** 2019-11-14

**Authors:** Yongyong Chen, Jing Su, Zirong Ren, Yongquan Huo

**Affiliations:** ^1^School of Education, Qinghai Normal University, Xining, China; ^2^School of Psychology, Shaanxi Normal University, Xining, China

**Keywords:** Tibetan undergraduates, minority students, optimism, mental health, cultural adaptability

## Abstract

Optimism, cultural adaptation, and mental health are distinct but associated concepts. An optimistic personality assists in maintaining mental health, and people with optimistic personality traits have better health than those with pessimistic personality traits. It has also been argued that (home or host) cultural factors influence the ability to adapt to individual social contexts and that interactions with individuals from different cultural backgrounds can help reduce social difficulties. Culture has a very important influence on the mental health of Tibetan college students, like other college students. This study aimed to investigate the initial mechanism of the potential influence of optimism on individuals’ mental health and cultural adaptability to/integration with mainstream culture. A total of 1027 Tibetan college students from four universities in western China were recruited for the study. The tools used included an instrument developed by the authors and used for the first time here to assess optimism, as well as the Depression Anxiety Stress Scales (DASS-21), Satisfaction with Life Scale (SWLS), and Positive Affect Scale (PAS). Optimism influenced mental health in the present study. The results were as follows: (1) cultural adaptability played a moderating role in the relationship between self-efficacy optimism and depression (β = 0.193, *p* < 0.01); (2) the moderating role of cultural adaptability in the relationship between optimism and positive emotions was not clear (*p* > 0.05); and (3) cultural adaptability moderated the relationship between self-efficacy optimism and life satisfaction (β = 0.286, *p* < 0.01). Thus, optimism and mental health are closely related, and cultural adaptability significantly affects the effect of self-efficacy in regulating depression and life satisfaction among Tibetan college students in China.

## Introduction

### Background

Acculturation has been a focus among anthropologists, sociologists, and psychologists. In a multicultural environment, the coexistence of mainstream and non-mainstream groups has become a common phenomenon, and interactions among different cultures is the new reality. Therefore, it is inevitable that non-mainstream groups and their members will experience acculturation under the influence of the dominating mainstream culture. Acculturation is thus an important theory in any cross-cultural scenario. It reflects the process of adapting to a new cultural environment, including changes in the attitude, behavior, and cognition of individuals or groups under a new environment (Tsai et al., 2002).

Acculturation includes both sociocultural and behavioral adaptation and is closely related to the mental state. Ward and Kennedy (1994) categorized acculturation into psychological and sociocultural adaptation and integration. Psychological adaptation primarily refers to psychological or emotional senses of happiness and satisfaction under new circumstances, whereas sociocultural adaptation refers to the ability to adapt or “acculturate” to the local sociocultural environment. Reduction in negative emotions, such as depression, anxiety, loneliness, despair, and homesickness, facilitates psychological adaptation.

The two most famous theories of cross-cultural adaptation are the “one-way model” and the “two-way model.” The one-way model refers to the abandonment of the old culture and adaptation to a new culture, whereas the two-way model uses two cultural orientations to describe cultural adaptation: the relationship with the original culture and the relationship with the (new) mainstream culture. Four types of cultural adaptation occur under this framework, namely, integration, separation, merging, and exclusion. Flannery et al. (2001) compared the one-way model with the two-way model through empirical research and proposed the three-way model, which includes the two dimensions of the two-way model as well as the new culture generated by the two coexisting cultural roles.

The cultural adaptation of Chinese ethnic groups is bidirectional. Mainstream ethnic culture (Han culture) and non-mainstream culture (ethnic minority culture) have mutual adaptation needs; that is, it is not just a matter of the ethnic minority culture adapting to Han culture. Therefore, the theoretical framework used in this study is Flannery’s three-dimensional model, in which the mainstream culture and minority culture are simultaneously independent and integrated, and on this basis, a new culture emerges.

This paper takes Tibetan students as the research object to explore whether social and cultural adaptation play a moderating role in the relationship between their optimism and mental health. China is a multiethnic country; Tibetans are one of the 55 ethnic minorities in China and are the aboriginal people of the Qinghai-Tibet Plateau. Tibetans are thus a cross-border ethnic group. China’s Tibetan areas include mainly the Qinghai-Tibet Plateau and the overlapping/surrounding areas, including the Tibet Autonomous Region, Qinghai, Sichuan, Gansu, and Yunnan, covering an area of 2.23 million square kilometers, or 23.31% of the national territory (Ran, 2003). Lhasa is a sacred place to the Tibetan people. According to China’s sixth census data (2011), the total Tibetan population is 6.28 million. As an important minority in China, the mental health level of Tibetan students undoubtedly impacts the overall mental health status of Chinese students.

The relationship between cultural adaptation and mental health has garnered considerable attention from researchers, and understanding this relationship is of great importance to the establishment of harmonious ethnic relations. Minority college students are the cultural elites of the Chinese minorities and serve as a bridge between minorities and the Han people. During their education, their levels of social and cultural adaptation and mental health are closely related to their growth and development.

Previous studies have focused on factors closely related to mental health but have ignored the psychological regulatory mechanisms related to these factors. Therefore, the present study explores whether cultural adaptation can explain the internal mechanism(s) of optimism and indicators of mental health, both negative (depression and anxiety) and positive (life satisfaction and positive emotions), in Tibetan college students in China to determine whether these individuals’ mental health can be improved by enhancing their optimism by fostering cultural adaptation.

### Impact of Chinese (Including Tibetan) College Students’ Optimism on Their Mental Health

Chinese society is undergoing rapid development, and the competition and challenges faced by college students are becoming more intense. Therefore, the mental health of college students in China has become a hot topic in psychology (Li, 2017). As strongly advocated by Seligman, the former president of the American Psychological Association, the “positive” movement in psychology has begun driving research in psychology toward positive mental qualities and positive mental health at the individual and population levels. Optimism is a major area in positive psychology research that involves the study of individuals’ positive experiences and attitudes toward the future. Tajfel and Turner (1979) argued that when an individual believes that his or her material or social prospects will bring him or her joy or benefit or meet the needs of society, this expectation engenders optimism. A great deal of research has been conducted on optimism in China and abroad (e.g., Hatchett and Park, 2004; Hu et al., 2012; Wei, 2014). Scheier and Carver (1992) argued that an optimistic individual generally holds a positive expectation of the future, which in turn brings positive energy to the individual, enabling him or her to handle and resolve problems in a positive way. Optimism is the core concept in positive psychology; it has been shown to bring many benefits to individuals, such as improved physical health, enhanced happiness, and success (Hao et al., 2016). Optimism and positive indicators of mental health are significantly and positively correlated (Zhou et al., 2015). In China, Yi (2012) showed that the correlation between optimism in college students and depressive symptoms was moderately negative and that the correlation between optimism and total daily stress was slightly negative.

Segerstrom et al. (1998) found that optimism is conducive to health maintenance and that, as a result, optimistic individuals are healthier than pessimistic individuals. In addition, Peterson et al. (1988) found that optimistic individuals do not become sick or seek medical attention as often as pessimistic individuals and have lower mortality rates. Optimistic individuals have not only stronger mental functions than pessimistic individuals (Achat et al., 2000) but also better health and positive emotions (Leventhal et al., 2003). Aspinwall and Brunhart (1996) revealed that optimistic individuals expect better things to happen in the future and are hopeful and confident; they also pay more attention to health-related information than pessimistic individuals do and thus are more likely to have healthier attitudes and behavior. Brissette et al. (2002) noted that optimistic individuals also have broader social networks and thus can more easily ask for help from friends when encountering challenges.

Optimism can significantly predict and promote mental health (Zhang et al., 2011; Zheng, 2012). In addition, in general, the higher an individual’s self-efficacy and optimism are, the healthier his or her behaviors (Posadzki et al., 2010). Thus, optimism in college students should be an important indicator of mental health and be considered crucial to their healthy mental growth.

### Previous Studies on the Indirect Impacts of Psychological and Cultural Adaptation

Previous research has shown that physical and mental health, which are affected by acculturation, act as direct or indirect indicators of acculturation level (Liu et al., 2014). Successful approaches to adaptation do not require complete abandonment of one’s own values and cultural identity, and the process of adaptation is not all-encompassing, such that minorities can continue to maintain their own values while integrating into a new culture (LaFromboise et al., 1993; Wong, 1999). Berry (1997) proposed that values, as a combination of cognition and beliefs that organize and guide an individual’s behavior, change when an individual adapts to a new environment and can thus determine the level of acculturation of college students of minority nationalities.

Both lateral and longitudinal research have indicated that stress from cross-cultural adaptation/integration is closely related to anxiety, depression, loneliness, and other problems. When individuals travel from one culture to another, they often experience gaps in information that is pertinent in the new context (Briones et al., 2012); this lack of information has unpredictable effects on behavior and creates stress for the individual. If such circumstances are handled improperly, individuals will experience more negative emotions, such as frustration, anxiety, and depression, reflecting adaptation difficulties and psychological stress. Adaptation stress may be a predisposing factor for anxiety, depression, and other problems in some but not all individuals engaging in cross-cultural adaptation; thus, individuals who bear the same stress do not necessarily experience the same psychological barriers. Therefore, there are likely moderating effects involved; in fact, the impact of adaptation stress on an individual’s psychological and sociocultural adaptation has been found to be restricted by the individual’s gender, personality, coping strategies for adaptation, social support networks, cultural adaptation strategies, and other factors (Yang and Clum, 1994; Zheng and David, 2003).

Although related studies have confirmed that there is a significant relationship between one’s acculturation attitude and cross-cultural adaptation results, the specific impacts of different acculturation strategies on an individual’s sociocultural and psychological adaptation have not yet been established. Kurman et al. (2005) showed a positive relationship between assimilation and sociocultural adaptation in ethnic minority groups, while Ward and Rana-Deuba (1999) suggested that travelers with assimilation strategies experienced fewer sociocultural adaptation problems and had higher sociocultural adaptation levels than other travelers. The findings of Wang and Mallinckrodt (2006) and Zhang and Goodson (2011) on Chinese international students studying in the United States were consistent with the previously reported findings: identification with one’s own culture (China) was not related to sociocultural adaptation, while identification with the host culture (the United States) allowed students to better adapt better, with fewer sociocultural adaptation problems.

The Tibetan college students in the present study had interacted with students from other cultures during their learning processes, creating cultural changes that inevitably impacted the Tibetan group and individuals but that likely had little impact on the Han group. For the Tibetan students, this impact might have penetrated into most or all aspects of their daily lives, causing cultural stress and psychological maladjustment or even personal crises in some individuals but possibly having a positive effect on others. Ward and Rana-Deuba (1999) proposed that individuals who tend to share a cultural identity with their native culture will experience better psychological adaptation, whereas individuals who tend to share a cultural identity with the host culture will experience significantly fewer social challenges. Mental health impacts college students’ values in life, perceptions, attitudes, language, and behavior, as well as their coping and adaptation strategies, which in turn impact culture.

### Significance of the Research

Cultural adaptation has been recognized as an important research field (Berry, 1980, 2006; Tadmor et al., 2009). International studies on cultural adaptation have usually focused on immigrants, refugees, and asylum-seekers, who are considered to have settled permanently in their new homes. However, the form of cultural adaptation explored in this paper is different. As a long-enduring minority in Chinese history, the Tibetan people experience different problems related to cultural adaptation than immigrants and refugees. When individuals of different ethnic groups come to an area where other ethnic groups live, they are cognitively prepared and behaviorally adaptable; the adaptation pressure brought by “cultural shock” is greatly reduced compared to that involved in the cultural adaptation situation of immigrants in Western countries, which has been the focus of previous cultural adaptation studies (Zhang, 2017). The cultural adaptation of the Tibetan people has been influenced by the culture and history of China and its environs for thousands of years; Tibetan subcultural elements have been incorporated into Chinese culture, and the two cultures have adapted to each other. In addition, the processes of immigrants’ adaptation to new cultural environments that have been studied internationally have been processes of one-way cultural adaptation. However, the cultures of all ethnic groups in China are bidirectional: minorities come to inland areas to study and work, and Han people also work and live in minority areas. There is a process of cultural adaptation in both situations (Zhang, 2017). Therefore, the study of the cultural adaptation of ethnic minorities in China has a unique cultural background that differs from that in Western countries. As a result, work such as the present research constitutes an expansion and enrichment of the research object and the content of cultural adaptation theory.

In most or all countries, ethnic minorities face acculturation problems due to cultural and political social changes. In a changing ecological environment, any human group will continuously cope with changes in survival and development. It is not that people cannot accept or adapt to change, but drastic change such as that associated with modernization can easily force people out of their normal routines or overwhelm them; coupled with the impact of developmental gaps, the relationships among different ethnicities are therefore deeply impacted (Sun, 2013). In a large, developing, multiethnic country sustaining strong economic growth, such as China, the acculturation of ethnic minority individuals, especially those living in multiethnic areas, thus attracts increasing interest.

The modes, processes, and results of acculturation will vary across groups and individuals within groups, even those living under the same environment. We should not only analyze the *current* state of life and social coexistence among the multiple ethnocultural groups in the Tibetan-inhabited areas of China to unveil their approaches to cultural self-adjustment but also, through the discussion of the mechanism of acculturation, try to further understand the complex *long-term* nature of the acculturation of ethnic minorities in relation to the goal of the construction of a harmonious multiethnic country. According to He and Mu (2013), “mutual contact, reliance, and interaction among ethnic groups do not lead to the disappearance of ethnic differences”; these authors argued that any ethnic group can absorb, abandon, change, reconstruct, and integrate new cultural factors by adapting to a changing external social environment, other cultures, and the mainstream culture to share a new pluralistic identity while maintaining and innovating within their native culture.

In a multicultural and multiethnic country, cultural adaptation poses an unavoidable challenge for minorities whose degree of cultural adaptation largely affects their development (Wu and Xu, 2015). When Tibetan students enter a new cultural environment from the cultural environment they are familiar with, they face problems related to cultural differences, which may give rise to anxiety or even affect their mental health (Bai, 2006). Optimism, as a stable psychological trait, plays an important role in promoting an individual’s social and cultural adaptation and mental health (Wang, 2013). Accordingly, the present study aimed to investigate the mental health of Tibetan students from the perspective of cultural adaptation and optimism.

Oberg et al. (2006) pointed out noted that cultural shock is intrinsic to acculturation and is often accompanied by negative psychological states such as helplessness, indecisiveness, low self-esteem, anxiety, or depression. Currently, ethnic Tibetans in China are undergoing great social, economic, and cultural changes, entailing intense “cultural shock” that impacts their mental health (Luo et al., 2009). Zheng and David (2003) showed that a high level of mainstream and minority cultural identity and cultural integration strategies are conducive to increasing individual life satisfaction and promoting psychological adaptation between cultures. Under different levels and stages of acculturation, individuals’ life satisfaction and mental health will thus vary. A thorough study of cultural adaptation in Tibetan college students moving to non-Tibetan areas of China will thus yield insights that will not only benefit their physical and mental health but also promote social stability. On this basis, the present study focuses on exploring sociocultural adaptation as a regulatory mechanism between optimism and mental health among Tibetan college students in non-Tibetan parts of China.

### Research Hypotheses

Intercultural adaptation is a dynamic and interactive process. Individuals influence their contexts and, in turn, are positively and negatively influenced by them. The role of cultural adaptation in the effect of optimism on life satisfaction is the focus of this study. Optimism affects mental health, as does cultural adaptation. Thus, we examine whether there is a relationship among these three variables.

Our hypotheses in the present study are as follows:

Hypothesis 1: There is a positive correlation between the optimism and mental health of Tibetan students in China, and optimism has a predictive effect on cultural adaptation and mental health.

Hypothesis 2: The cultural adaptation of Tibetan students in China is related to their mental health.

Hypothesis 3: Cultural adaptation plays a moderating role in the effect of optimism on the mental health of Tibetan students in China.

## Materials and Methods

### Participants

In total, 1200 questionnaires were distributed at Qinghai University for Nationalities, Nationalities Institute of Qinghai Normal University, Northwest University for Nationalities, and Tibet University, which are four higher education institutions in Qinghai, Gansu, and Tibet. A total of 1027 valid questionnaire responses were received, for an 85.58% effective response rate. Of the students, 46.45% were male, and 53.55% were female; 26% were freshmen, 32.72% were sophomores, 22.20% were juniors, and 19.08% were seniors. In addition, 40.80% were arts students, and 59.20% were science students; 18.80% were from pastoral areas, 61.83% were from farming areas, 5.74% were from towns, 8.37% were from county-level municipalities, and 5.26% were from cities.

According to a survey conducted in the mid-1980s, a large proportion of the Tibetan population in China lives in urban and rural areas, and a small proportion lives in pastoral areas (Lu, 2012). In China, a county-level city is a city established to supplant a county; it is an urbanizing administrative entity that defines the original county as an urban area and turns the surrounding towns into village centers (Wang et al., 2009).

Since 1978, three patterns of primary and secondary education have been applied in Tibetan areas. The “first model” is taught mainly in the Tibetan language, plus some Chinese. The students who are taught under this model come mainly from farming and pastoral areas and do not understand Chinese or have poor Chinese proficiency. The “second model” is taught mainly in Chinese, plus some Tibetan. The students who are taught under this model are mainly urban Tibetan students who have some Chinese language foundation. In the “third model,” students do not learn the Tibetan language. They are mainly Tibetan but also include students of Han and other ethnicities, and they have a good Chinese foundation.

The average age of the sample was 20.31 years (SD = 1.85). In terms of the educational background of respondents’ fathers, 19.09% had not graduated elementary school, 52.19% had graduated elementary school, 21.03% had graduated high school or technical secondary school, and 7.69% had graduated from universities or graduate school. In terms of the educational background of respondents’ mothers, 41.09% were illiterate, 45.76% had graduated elementary school, 9.54% had graduated high school or technical secondary school, and 3.6% had graduated from university or graduate school. In total, 88.31% of the Tibetan students lived mainly among Tibetans, 6.04% lived mainly among Han Chinese, and 5.65% lived among an essentially even distribution of Tibetans and Han.

### Measures

In this study, gender, academic year, home location, and surrounding population were taken as the control variables. Gender: The incidence of depression tends to be not only gender-specific but also situational. The risk factors related to depression are also different among different gender groups (Tao, 2006). Academic year: Most studies of college students have taken the year or “grade” as a control variable. Previous research on college students’ optimism and mental health has found significant differences in overall levels of optimism among different grade levels (Yu and Zheng, 2011). Home location: Home locations mainly include towns, agricultural areas, and pastoral areas. Differences in regional factors relate to significant differences in cultural adaptation. In a study of cultural adaptation among farmers in Jiangsu Province, Ye (2011) found significant differences in the family conditions of students from different places, which produced differences in students’ cultural adaptation to college life. Surrounding population: In the adaptation of migrant children in Beijing, especially those from agricultural and pastoral areas, differences between the surrounding population in source and target areas have been shown to significantly affect mental health (Yuan et al., 2009).

#### Life Orientation Test-Revised for Tibetan Undergraduates

A tool was developed by one of the present authors to assess Tibetan college students’ optimism (Chen and Huo, 2015). The tool was thoroughly validated: the eigenvalue of questionnaire dimension 1 (optimistic tendency) was 4.620, and the contribution rate was 20.186%; the eigenvalue of dimension 2 (pessimistic tendency) was 3.552, and the contribution rate was 16.097%; and the eigenvalue of dimension 3 (self-efficacy optimism) was 2.846, and the contribution rate was 10.024%. The total contribution rate of the optimism questionnaire was 46.307%.

There are 23 questions in the questionnaire. Exploratory factor analysis of the questionnaire showed that factor 1 was composed of 7 questions that were mainly related to an optimistic view and the expectation that events would develop in a favorable direction; hence, this factor was named “optimistic tendency.” Factor 2 was composed of 6 questions that were mainly related to a pessimistic view and the expectation that events would develop in an unfavorable direction; thus, this factor was named “pessimistic tendency.” Factor 3 consisted of 10 questions that were related to the tendency to positively evaluate individual abilities and behaviors and were not related to past experiences and behaviors; this factor was therefore named “self-efficacy optimism.”

The correlation among the three factors of the optimism questionnaire was moderate, while the correlation between the three factors and the total score of the questionnaire was high and significant. Optimistic tendency and self-efficacy optimism were significantly positively correlated with the total score; the correlation coefficients were *r* = 0.081 and *r* = 0.905, respectively, *p* < 0.01. Pessimistic tendency was significantly negatively correlated with total score; the correlation coefficient was *r* = −0.558, *p* < 0.01. Optimistic tendency and self-efficacy optimism were negatively correlated with pessimistic tendency; the correlation coefficients were *r* = −0.152 and *r* = −0.298, respectively, *p* < 0.01. Optimistic tendency was moderately and positively correlated with self-efficacy optimism; the correlation coefficient was *r* = 0.548, *p* < 0.01. This result shows that the three factors of the optimism questionnaire were independent but moderately correlated. The tool had good discriminatory validity and aggregation validity, indicating overall construct validity.

Thus, the questionnaire contains three dimensions—optimistic tendency, pessimistic tendency, and self-efficacy optimism; a 5-point Likert scale was used to assess the scores (from “strongly disagree” to “strongly agree”). The Cronbach’s alpha coefficient of optimistic tendency was 0.706, and its split-half reliability was 0.672; the Cronbach’s alpha of pessimistic tendency was 0.760, and its split-half reliability was 0.751; and the Cronbach’s alpha of self-efficacy optimism was 0.815, and its split-half reliability was 0.762. The coefficient of internal consistency of the full questionnaire reached 0.835, and the split-half reliability was 0.787; 4 weeks later, we carried out a retest with 62 Tibetan students to calculate the test–retest reliability, and the coefficient of internal consistency was 0.643, showing that the questionnaire had good reliability. The results of the confirmatory factor analysis showed that χ^2^/df = 4.06, GFI = 0.87, AGFI = 0.91, RMSEA = 0.071, CFI = 0.93, and RMR = 0.082, indicating that the three-factor model fit the data well and that the validity of the concept was good. Thus, this questionnaire can be used as a tool for researching optimism in Tibetan college students in China (Chen and Huo, 2015).

#### Brief Version of the Depression Anxiety Stress Scales (DASS-21)

We used the Chinese revised version of the DASS-21 by Gong et al. (2010). In the DASS-21, each dimension (repression, anxiety, and stress) contains seven items; the coefficient of internal consistency was 0.79, while the coefficient of internal consistency for the total scale was 0.89. The DASS-21 has good construct validity and criterion-related validity. In this study, the Cronbach’s alpha coefficient of the scale was 0.88.

#### Satisfaction With Life Scale

The SWLS, developed by Diener et al. (1985), includes five items to test university students’ satisfaction with life. This scale uses a 7-point Likert scoring format (from “strongly disagree” to “strongly agree”). The average score of five items is calculated; the higher the score, the more satisfied students are (Diener et al., 1985). Pavot et al. (1991) found that the internal consistency reliability coefficient for the SWLS was 0.87, and 2 months later, the coefficient was 0.82, with good criterion validity. Moreover, the correlation between the SWLS and other subjective health scores indicates that this scale has good content validity (Pavot and Diener, 1993). In addition, the Cronbach’s alpha for internal consistency reliability was 0.74 (Yuan, 2005); in this study, the Cronbach’s alpha coefficient of the scale was 0.84.

#### Positive Affect Scale

This 10-item subscale is based on the positive emotion scale prepared by Watson et al. (1988). Emotions are rated on a 5-point Likert-type scale ranging from “none” to “extreme”; the higher the score is, the more positive emotions that are present. Huang et al. (2003) showed that the interrater consistency for the positive emotion scale was 0.85, which means that the PAS has good reliability. Moreover, the structure of the Chinese version is in accordance with the English version, with good construct validity as well as criterion validity. In this study, the Cronbach’s alpha coefficient of the scale was 0.88. As the PAS is a self-report measure, we also calculated the kappa coefficient. Cohen’s kappa is a widely used indicator of scoring consistency among assessors (Cohen, 1960) to correct accidental consistency. The kappa value in this study was 0.80; according to Landis and Koch (1977), a kappa value in the range of 0.61–0.80 is considered to indicate “greater” consistency, and a kappa value over 0.81 is considered to indicate “almost perfect” consistency. Therefore, in this study, the PAS was corrected for contingency consistency, and the consistency was found to be ideal.

#### Social Cultural Adaptability Scale

We used the Social Cultural Adaptability Scale, developed by Ward and Rana-Deuba (1999) and revised by Zhang et al. (2008), which assesses problems in learning, life, and communication. The dimension of interpersonal behavior refers to the tendency of minority students to seek communication with other groups in other cultures; the material life dimension refers to the ability of minority students to adapt to an unexpected living environment and culture different from their original culture and living area; and the cognitive value dimension refers to the ability of minority students to understand and accept the differences between different cultural groups. The cumulative variance contribution rate of these three dimensions was 47.38%. The factor loading matrix showed that the loadings of the other factors exceeded 0.50, except for items 4, 11, 14, and 24. Based on principal component analysis, items with factor loadings of less than 0.3 and fewer than 1 feature were deleted. Items 4, 11, 14, and 24 were kept as variables for the Social Cultural Adaptability Scale, with loadings of 0.481, 0.467, 0.493, and 0.459, respectively. The scale has good structural validity.

This scale includes 26 items with a 5-point Likert-type rating scale (from 1 = “without difficulty” to 5 = “great difficulty”); the higher the score is, the more difficulty the individual has adapting to the culture. The Cronbach’s alpha for the material life factor was 0.850, with 0.679 split-half reliability; the Cronbach’s alpha for the interpersonal behavior factor was 0.868, with 0.665 split-half reliability; and the Cronbach’s alpha for the perceived value factor was 0.859, with 0.783 split-half reliability. Four weeks later, we carried out a retest with 62 Tibetan undergraduates, and the reliability was 0.876, showing that the questionnaire was in accordance with the measurement standard and could be used to measure Tibetan undergraduates’ social cultural adaptability. In this study, the Cronbach’s alpha coefficient of the scale was 0.86.

#### Common Method Bias Test

Harman’s single-factor test was used to assess the common method bias of the data studied (Kolar and Zabkar, 2010). According to the hypothesis of the single-factor model, when all the items were loaded onto one factor, the fit was worse than that of the original model (χ^2^ = 16637.866, DF = 307, *p* = 0.00, RMSEA = 0.121, GFI = 0.109, AGFI = 0.117, RMR = 0.230, CFI = 0.533). Therefore, there was no common method deviation in the data used in this study.

### Procedure

The testers in the present study (who implemented the questionnaires) were ethnopsychology and developmental psychology graduate students who had experience conducting surveys. All testers were provided training on the instruction of respondents in how to complete the questionnaire, the questionnaire content, and precautionary items. The questionnaire was given in class groups, which were each overseen by 2–3 testers. First, testers read the instructions and material on the purpose of the test, answer format, optional questions, and anonymity/privacy. Then, participants were required to complete the test independently within the specified time (approximately 30 min); questionnaires were collected immediately.

In this survey, 1200 questionnaires were distributed, from which 173 invalid questionnaires were excluded; among these invalid questionnaires, 131 were incomplete, 28 were not completed, and 14 had been answered with all of the same answers. Excluding these invalid questionnaires, 1027 valid questionnaires remained. These data were analyzed using SPSS 19.0.

## Results

### Analyses of the Principal Variables

Pearson correlations were used to quantify the relationships between optimistic tendency, pessimistic tendency, self-efficacy optimism, social cultural adaptability (income, interpersonal behavior, recognition value), and mental health (see Table 1). The depression and anxiety subscales of DASS-21 were used as negative mental health indicators: the higher the score was, the higher the emotion. The questions explored the relationship between pessimism and depression. In our research, pessimism is different from depression: a pessimistic tendency refers to pessimistic attitudes toward the present and future, meaning that people with pessimistic tendencies have negative expectations of what will happen in the future; they always see the negative side of things and think that events will always develop in an unfavorable direction.

**TABLE 1:** Correlation analysis between variables.

	**1**	**2**	**3**	**4**	**5**	**6**	**7**	**8**	**9**	**10**	**11**
1. Optimistic tendency	–										
2. Pessimistic tendency	−0.099^∗^	–									
3. Self-efficacy optimism	0.530^∗∗∗^	–0.321^∗∗∗^	–								
4. Anxiety	–0.234^∗∗^	0.312^∗∗∗^	−0.072^∗^	–							
5. Depression	–0.140^∗∗^	0.320^∗∗∗^	−0.096^∗^	0.535^∗∗∗^	–						
6. Stress	–0.281^∗∗^	0.307^∗∗∗^	–0.001	0.481^∗∗∗^	0.805^∗∗∗^	–					
7. Life satisfaction	0.323^∗∗∗^	−0.061^∗^	0.331^∗∗∗^	–0.144^∗∗^	–0.150^∗∗^	–0.110^∗∗^	–				
8. Positive emotions	0.346^∗∗∗^	−0.093^∗^	0.443^∗∗∗^	–0.029	0.010	–0.021	0.426^∗∗^	–			
9. Income	0.065^∗^	–0.190^∗∗^	–0.040	–0.246^∗∗^	–0.228^∗∗^	0.240^∗∗^	0.092^∗^	–0.011	–		
10. Interpersonal behavior	0.126^∗∗^	–0.186^∗∗^	–0.020	–0.296^∗∗^	–0.271^∗∗^	0.234^∗∗^	0.082^∗^	–0.003	0.552^∗∗∗^	–	
11. Recognition value	0.105^∗∗^	–0.171^∗∗^	−0.054^∗^	–0.216^∗∗^	–0.201^∗∗^	0.168^∗∗^	0.115^∗∗^	–0.003	0.454^∗∗∗^	0.318^∗∗^	–
*M*	3.611	2.982	3.327	0.669	0.745	0.881	4.261	3.108	2.402	2.145	2.272
SD	0.555	0.551	0.541	0.585	0.565	0.525	1.140	0.608	0.769	0.824	0.748

*^∗^*p* < 0.05, ^∗∗^*p* < 0.01, and ^∗∗∗^*p* < 0.001.*

Scores from the PAS and SWLS were combined to form a positive mental health index, while the DASS depression and stress scores were combined to form a negative mental health index. Optimism was significantly positively related to the positive mental health index (SWLS *r* = 0.32, PAS *r* = 0.35, *p* < 0.001) and significantly negatively related to the negative mental health index (Depression *r* = −0.14, Anxiety *r* = −0.23, *p* < 0.01). Pessimistic tendency was negatively related to the positive mental health index (SWLS *r* = −0.06, PAS *r* = −0.09, *p* < 0.05) and significantly positively related to the negative mental health index (Depression *r* = 0.32, Anxiety *r* = 0.31, *p* < 0.001). Self-efficacy optimism was significantly positively related to the positive mental health index (SWLS *r* = 0.33, PAS *r* = 0.44, *p* < 0.001) and negatively related to the negative mental health index (Depression *r* = −0.10, Anxiety *r* = −0.07, *p* < 0.05).

In terms of cultural adaptability, income, interpersonal behavior, recognition value’ and optimistic tendency were significantly positively related to each other (*r* = 0.07, *p* < 0.05; *r* = 0.13, *p* < 0.01; *r* = 0.11, *p* < 0.01), negatively related to pessimistic tendency (*r* = −0.19, *r* = −0.19, *r* = −0.17, respectively; *ps* < 0.01), income and interpersonal behavior were unrelated to self-efficacy optimism (*ps* > *0.05*), recognition value was negatively related to self-efficacy optimism (*r* = −0.06, *p* < 0.05). Additionally, these three dimensions were associated with the depression component of the negative mental health index (*r* = −0.23, *r* = −0.27, *r* = −0.20, respectively; *ps* < 0.01), were significantly negatively related to anxiety (*r* = −0.25, *r* = −0.30, *r* = −0.22, respectively; *ps* < 0.01), were significantly positively related to the positive mental health index and life satisfaction (*r* = 0.09, *p* < 0.05; *r* = 0.08, *p* < 0.05; *r* = 0.12, *p* < 0.01), and were not significantly related to positive emotions.

These results reflect associations that could have been affected by the demographic variables. Therefore, we subsequently adjusted for the demographic variables using regression analysis to explore the relationship between optimism and mental health and to highlight the moderating effects of cultural adaptability and stress.

### Moderating Effect of Cultural Adaptability

#### Moderating Effect of Cultural Adaptability on the Relationship Between Optimism and Negative Dimensions

When the relationship between a dependent variable (Y) and an independent variable (X) is affected by a third variable (M), then M should be considered a moderator variable. In this study, optimistic tendency, pessimistic tendency, self-efficacy optimism, social and cultural adaptability (income, interpersonal behavior, cognitive value), and mental health were all continuous variables. Wen et al. (2005) proposed a hierarchical regression analysis for cases where both independent variables and regulatory variables are continuous variables. Therefore, a stratified regression method was used in the analysis of regulatory effects. We used hierarchical regression to explore moderating effects and adjust for the demographic variables as dummy variables.

Gender, grade, home location, and surrounding people (such as school friends) may lead to depression. The correlation between optimism and mental health also varies across different cultural backgrounds (Qi et al., 2012). One study showed that the optimism of freshmen was significantly higher than that of sophomores and juniors, even when there was no significant difference in professional orientation between them (Jiang, 2008). Another study showed no significant gender difference in overall levels of optimism, but male respondents’ positive attitudes and positive face scores were significantly higher than those of female respondents, and females’ acceptable reality scores were significantly higher than those of males (Yu and Zheng, 2011).

Therefore, we selected these variables as the first set of control variables. Table 2 shows that the control variables explained 6.1% of the variance in depression [*F*(1, 126) = 13.01, *p* < 0.001]; among the variables, gender and home location were significant predictors (β = −0.09, β = 0.05, respectively; *ps* < 0.05), as was grade (β = 0.11, *p* < 0.001). Female respondents were more likely to be depressed than males, and students who lived in cities where their families lived were more likely to be depressed. Optimistic tendency, pessimistic tendency, and self-efficacy optimism were introduced into the model in the second step; adding these variables explained 14.9% of the variance in depression [*F*(1, 950) = 33.08, *p* < 0.001]. Optimistic tendency and self-efficacy optimism were significant negative predictors of depression (β = −0.25, *p* < 0.001; β = −0.21, *p* < 0.01), while pessimistic tendency was a significant positive predictor of depression (β = 0.36, *p* < 0.001). In the third step, the moderating cultural variables were introduced into the model; the results showed that cultural adaptability was a significant negative predictor of depression (β = −0.17, *p* < 0.001). In the fourth step, the appropriate three subscales of optimism and cultural adaptability were entered into the model; the product increased the amount of variance explained in depression by 2.4% [*F*(1, 932) = 31.26, *p* < 0.001]. Self-efficacy optimism was significantly related to the product of cultural adaptability (β = 0.19, *p* < 0.01), demonstrating that cultural adaptability played a positive role in moderating the relationship between self-efficacy and depression.

**TABLE 2:** Moderating effect of cultural adaptability on the relation between optimism and depression.

**Variable**	**Depression**			
	**First model**	**Second model**	**Third model**	**Fourth model**
**Step 1: control variables**				
Gender	−0.093^∗^	–0.090^∗∗^	–0.109^∗∗^	–0.108^∗∗^
Grade	0.105^∗∗∗^	0.089^∗∗∗^	0.082^∗∗∗^	0.089^∗∗∗^
Home location	0.045^∗^	0.045^∗∗∗^	0.053^∗∗^	0.050^∗∗^
Surrounding people	0.051	0.030	0.013	0.010
**Step 2: independent variables**				
Optimistic tendency		–0.248^∗∗∗^	–0.215^∗∗∗^	–0.086
Pessimistic tendency		0.355^∗∗∗^	0.312^∗∗∗^	0.223^∗^
Self-efficacy optimism		–0.207^∗∗^	–0.104^∗∗^	–0.437^∗∗^
**Step 3: moderating variables**				
Cultural adaptability			–0.172^∗∗∗^	–0.910^∗∗∗^
**Step 4: independent variables^∗^regulatory variables**				
Optimistic tendency^∗^cultural adaptability				0.057
Pessimistic tendency^∗^cultural adaptability				–0.046
Self-efficacy optimism^∗^cultural adaptability				0.193^∗∗^
*F*	13.013^∗∗∗^	33.081^∗∗∗^	36.901^∗∗∗^	31.259^∗∗∗^
*R*^2^	0.061	0.210	0.251	0.275
Δ*R*^2^	0.061	0.149^∗∗∗^	0.040^∗∗∗^	0.024^∗∗∗^

*^∗^*p* < 0.05, ^∗∗^*p* < 0.01, and ^∗∗∗^*p* < 0.001.*

**FIGURE 1:**
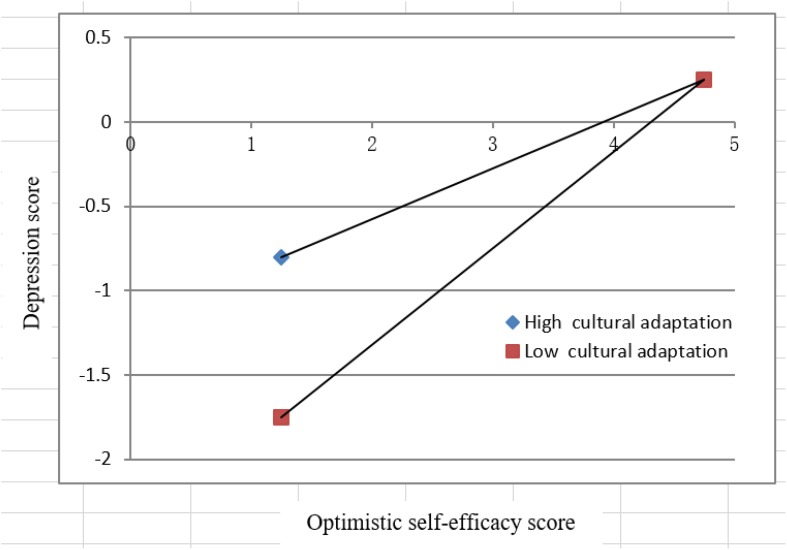
Moderating effects of cultural adaptability on the relation between self-efficacy optimism and depression.

To further explain the regulating effect between self-efficacy and depression, participants were divided into two groups: a group with high scores on self-efficacy optimism and cultural adaptability (higher than the average standard score) and a group with low scores (lower than the average standard score). Figure 1 shows that poor cultural adaptability was negatively related to depression; that is, the lower the self-efficacy optimism score was, the more likely respondents were to be depressed.

Table 3 shows that optimistic tendency, pessimistic tendency, and self-efficacy optimism were not significantly related (*p* > 0.01), indicating that cultural adaptability did not affect the relationship between optimism and anxiety.

**TABLE 3:** Moderating effect of cultural adaptability on the relation between optimism and anxiety.

**Variable**	**Anxiety**			
	**First model**	**Second model**	**Third model**	**Fourth model**
**Step 1: control variables**				
Gender	–0.049	–0.046	–0.063	–0.063
Grade	0.093^∗∗∗^	0.081^∗∗∗^	0.075^∗∗∗^	0.078^∗∗∗^
Home location	0.024	0.028	0.035	0.033
Surrounding people	0.037	0.017	0.002	0.002
**Step 2: independent variables**				
Optimistic tendency		–0.156^∗∗∗^	–0.127^∗∗^	0.056
Pessimistic tendency		0.337^∗∗∗^	0.298^∗∗∗^	0.264^∗∗^
Self-efficacy optimism		0.017	0.015	0.158
**Step 3: regulatory variables**				
Cultural adaptability			–0.151^∗∗∗^	0.614^∗∗^
**Step 4: independent variables^∗^regulatory variables**				
Optimistic tendency^∗^cultural adaptability				–0.080
Pessimistic tendency^∗^cultural adaptability				0.019
Self-efficacy optimism^∗^cultural adaptability				–0.066
*F*	8.038^∗∗∗^	23.368^∗∗∗^	26.160^∗∗∗^	20.662^∗∗∗^
*R*^2^	0.039	0.158	0.192	0.200
Δ*R*^2^	0.039	0.120^∗∗∗^	0.033^∗∗∗^	0.009^∗^

*^∗^*p* < 0.05, ^∗∗^*p* < 0.01, and ^∗∗∗^*p* < 0.001.*

#### Moderating Effect of Cultural Adaptability on the Relationship Between Optimism and Positive Dimensions

Table 4 shows that cultural adaptability regulated the relationship between self-efficacy optimism and life satisfaction.

**TABLE 4:** Moderating effect of cultural adaptability on the relation between self-efficacy optimism and life satisfaction.

**Variable**	**Life satisfaction**			
	**First model**	**Second model**	**Third model**	**Fourth model**
**Step 1: control variables**				
Gender	0.014	0.033	0.042	0.043
Grade	0.004	–0.001	0.003	–0.002
Home location	0.000	0.027	0.024	0.026
Surrounding people	–0.057	–0.035	–0.027	–0.024
**Step 2: independent variables**				
Optimistic tendency		0.373^∗∗∗^	0.357^∗∗∗^	0.666^∗^
Pessimistic tendency		–0.262^∗∗∗^	–0.241^∗∗∗^	–0.272
Self-efficacy optimism		0.551^∗∗∗^	0.553^∗∗∗^	–0.112
**Step 3: regulatory variables**				
Cultural adaptability			0.182^∗∗∗^	–0.630
**Step 4: independent variables^∗^regulatory variables**				
Optimistic tendency^∗^cultural adaptability				–0.135
Pessimistic tendency^∗^cultural adaptability				0.016
Self-efficacy optimism^∗^cultural adaptability				0.286^∗∗^
*F*	0.251^∗∗∗^	21.362^∗∗∗^	19.348^∗∗∗^	15.192^∗∗∗^
*R*^2^	0.012	0.147	0.149	0.156
Δ*R*^2^	0.012	0.145^∗∗∗^	0.002	0.006

*^∗^*p* < 0.05, ^∗∗^*p* < 0.01, and ^∗∗∗^*p* < 0.001.*

**FIGURE 2:**
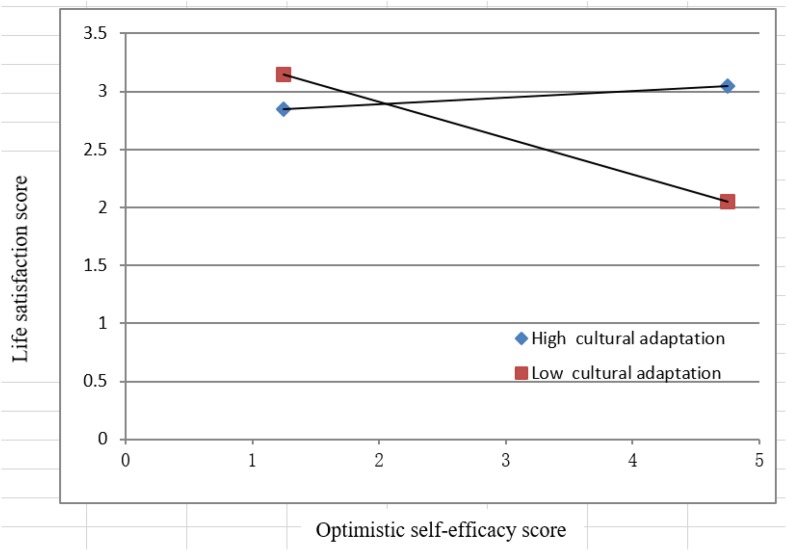
Moderating effects of cultural adaptability on the relation between self-efficacy optimism and life satisfaction.

The interaction effect shown in Figure 2 indicates that among participants with poorer cultural adaptability, self-efficacy optimism had a more positive effect on life satisfaction.

Table 5 shows that cultural adaptability did not significantly moderate the relationship between optimism and positive emotions.

**TABLE 5:** Moderating effect of cultural adaptability on the relation between optimism and positive emotion.

**Variable**	**Positive emotion**			
	**First model**	**Second model**	**Third model**	**Fourth model**
**Step 1: control variables**				
Gender	–0.160^∗∗∗^	–0.139^∗∗∗^	–0.143^∗∗∗^	–0.142^∗∗∗^
Grade	0.029	0.014	0.013	0.011
Home location	0.013	0.028	0.030	0.031
Surrounding people	0.018	0.029	0.026	0.020
**Step 2: independent variables**				
Optimistic tendency		0.095^∗^	0.102^∗^	–0.096
Pessimistic tendency		–0.127^∗∗∗^	–0.135^∗∗∗^	–0.288^∗∗^
Self-efficacy optimism		0.496^∗∗∗^	0.496^∗∗∗^	0.566^∗∗∗^
**Step 3: regulatory variables**				
cultural adaptability			0.233^∗∗∗^	−0.387^∗^
**Step 4: independent variable^∗^ regulatory variables**				
Optimistic tendency^∗^ cultural adaptability				0.085
Pessimistic tendency^∗^ cultural adaptability				0.069
Self-efficacy optimism^∗^ cultural adaptability				–0.031
*F*	5.927^∗∗∗^	39.662^∗∗∗^	35.478^∗∗∗^	27.122^∗∗∗^
*R*^2^	0.029	0.242	0.244	0.248
Δ*R*^2^	0.029	0.213^∗∗∗^	0.001	0.004

*^∗^*p* < 0.05, ^∗∗^*p* < 0.01, and ^∗∗∗^*p* < 0.001.*

## Discussion

Studies on the relationship between social and cultural adaptation for the mental health of Tibetan college students have shown that those with better social and cultural adaptation have better mental health and vice versa (Luo et al., 2011).

The present study used cultural adaptation as a variable to explore the relationship between the optimism and mental health of Tibetan college students and examined the impact of cultural adaptation on their optimism and mental health.

### Moderating Effect of Sociocultural Adaptation on the Relationship Between Optimism and Depression in Tibetan College Students

The present study validated the positive predictive effect of optimism on mental health (Scheier and Carver, 1992; Posadzki et al., 2010; Zhang et al., 2011; Zheng, 2012; Qi, 2013). Zhang and Goodson (2011) studied 508 Chinese international students in the United States; the findings showed that the adoption of an integration strategy in relation to the host culture had a significantly negative relationship with depression. Yi (2012) indicated that the correlation between optimism and depression symptoms in college students was moderately negative, while that between optimism and social understanding was moderately positive and that between optimism and stressful incidents in daily life was slightly negative.

In Figures 1, 2, under low and high cultural adaptation, the relationship between self-efficacy optimism and depression scores shows an inconsistent trend, which means there is a moderating effect of cultural adaptation between the two variables. When self-efficacy optimism is low, the depression score under low cultural adaptation is higher than that under high cultural adaptation; as the self-efficacy optimism score increases, the gap between depression scores under high and low cultural adaptation narrows. Depression results from the interaction of biological, psychological, and social (cultural) factors, with contributing factors such as previous life experiences and cultural background. Self-efficacy optimism does not involve factors such as previous experience and cultural beliefs, whereas cultural adaptation does involve factors such as original cultural background and cultural beliefs. When cultural adaptation is added as a moderating variable to examine the impact of self-efficacy optimism on depression, the original influence path will change and thus trigger a moderating effect.

### Moderating Effect of Sociocultural Adaptation on the Relationship Between Optimism and Anxiety in Tibetan College Students

The present study found that sociocultural adaptation does not have a significant moderating effect on the relationship between optimism and anxiety in Tibetan college students. This result is consistent with the findings of Wang and Mallinckrodt (2006) that showed that students’ identification with their native culture (Chinese culture) was not significantly correlated with individual anxiety.

In a study of minority students in Ethiopia, a slight positive correlation was found between positive attitudes toward the mainstream cultural group and psychological adaptation. In addition, Kurman et al. (2005) found that among minority students in Israel, the impact of perceptions of the mainstream group’s attitude toward the minority group outweighed the impact of acculturation attitudes on anxiety but did not outweigh the impact of social adaptation. The findings of the present study are thus consistent with those of previous studies. For college students, anxiety is a nervous, frustrating, and fearful emotional state experienced when dealing with current or future events – it may include test anxiety, employment anxiety, or interpersonal anxiety. The time factor of anxiety is in the future, which means it is less related previous life experiences (Mu et al., 2004). Social and cultural adaptation encompasses the impact of different cultural beliefs on the current situation, which in turn involves past experiences. There are significant differences between anxiety and social and cultural adaptation. The regulatory effect of social and cultural adaptation is not significant, probably because the moderating effects of past experiences and social and cultural adaptation cannot change the impact of optimism on anxiety.

### Moderating Effect of Sociocultural Adaptation on the Relationship Between Optimism and Life Satisfaction in Tibetan College Students

Sociocultural adaptation has a significant moderating effect on the relationship between self-efficacy optimism and life satisfaction in Tibetan college students, which is consistent with the findings of Zhang et al. (2008). Students who scored high in optimism had higher adaptability and better mental health, and there was an interaction between optimism and adaptability. Qi (2013) revealed that college students’ adaptability and optimism had significantly positive effects on life satisfaction; that is, the more adaptive students were and the better their temperamental optimism was, the higher their life satisfaction. Qin and Piao (2011) further found that temperamental optimism had a direct, significant effect on the life satisfaction of Japanese students living outside Japan.

As shown in Figure 2, when the self-efficacy optimism score is low, the life satisfaction score under low cultural adaptation is higher than that under high cultural adaptation, while when the self-efficacy optimism score is high, the life satisfaction score under high cultural adaptation is higher than that under low cultural adaptation. Life satisfaction is an overall cognitive assessment of an individual’s living conditions most of the time or for a certain period of time and is again determined by factors such as previous life experiences and cultural background. Self-efficacy optimism, in contrast, does not involve factors such as past experiences or subjective destiny, while cultural adaptation is affected by factors such as original cultural background and cultural beliefs. When cultural adaptation is added to the relationship between self-efficacy optimism and life satisfaction, it changes the original influence path; that is, it triggers a moderating effect.

### Research Limitations and Outlook

#### Research Limitations

Some limitations may impact the validity of the study. First, the present study adopted a psychometric data collection approach. Because of the limitations of the measurement method, the data collected may have been subject to common method variance, and subjects’ responses may have been affected by a social gratitude effect, that is, by the need for appreciation and acceptance and the belief that cultural acceptance and approval could satisfy this need. To minimize the potential adverse effects of the method, the “Questionnaire on Tibetan College Students’ Optimism” was developed. Exploratory factor analysis and confirmatory factor analysis were adopted, and the validity of the questionnaire was found to be high. Subsequent research may use longitudinal data analysis to improve the research design and further verify the results.

Second, the impact of cultural adaptability on Tibetan college students’ mental health needs to be further studied, as the present study investigated only its moderating effect on the relationship between optimism and mental health. Future studies are needed to incorporate other core factors.

Finally, in future studies, we suggest that Johnson and Neyman’s method be adopted to test simple slopes when the moderator is a continuous variable and that the pick-a-point method be adopted to test simple slopes when the moderator is a categorical variable or when researchers are interested in testing some special value of the moderator.

#### Research Implications and Outlook

This study explored the relationship between optimism, mental health, and cultural adaptation among Tibetan students in China. It has strong theoretical significance and reference value, as follows.

First, this study created an optimism scale and revised a cultural adaptation scale for Tibetan students in China, providing a reliable measurement tool for studying Tibetan students’ optimism and cultural adaptation. More generally, this study provides a reference for research on positive psychological qualities and cultural adaptation among ethnic minorities in China.

Second, this study constructed a moderation model of optimism, mental health, and cultural adaptation for Tibetan students in China through quantitative research and empirically tested the relationships between all parts of the model. Starting from primary materials, this study explored and established a relationship model in the sample analysis, laying a foundation for follow-up research.

Finally, since China is a multiethnic country, the study findings can stimulate thinking about positive psychological qualities and cultural adaptation among Chinese minorities, which can in turn help draw more research attention to the mental health problems brought about by cultural adaptation.

In future analyses of acculturation, identity attitudes and separation attitudes of mainstream cultural groups should not be overlooked; in-depth analysis of these topics is warranted.

## Conclusion

The main conclusions of this study are as follows:

(1) There are differences in optimism, cultural adaptation, and mental health among Tibetan students in China related to gender, academic specialty, academic year, place of origin, parents’ educational level, and the surrounding population.
(2) There is a positive correlation between the optimism and mental health of Tibetan students in non-Tibetan parts of China, and optimism has a predictive effect on cultural adaptation and mental health.
(3) The cultural adaptation of Tibetan students in non-Tibetan parts of China is related to their mental health.
(4) Cultural adaptation plays a moderating role in the effect of optimism on the mental health of Tibetan students in non-Tibetan parts of China.

## Ethics Statement

The study was approved by the academic and ethics committee of the School of Education, Qinghai Normal University, affiliated with the Academic Ethics Committee of Qinghai Normal University. The academic and ethics committees approved the consent procedure. All procedures performed in studies involving human participants were in accordance with the ethical standards of the institutional and/or national research committee and with the 1964 Helsinki declaration and its later amendments or comparable ethical standards. Informed consent was obtained from all individual participants included in the study. The consent obtained from all participants was both written and informed.

## Author Contributions

YC proposed the research ideas, designed the research programs, analyzed the data, and was responsible for drafting the manuscript and revising the final version. JS was responsible for collecting the data and analyzed the data. ZR was responsible for collection, data analysis, and manuscript revision. YH reviewed the manuscript and proposed some constructive suggestions for modification.

## Conflict of Interest

The authors declare that the research was conducted in the absence of any commercial or financial relationships that could be construed as a potential conflict of interest.
